# MicroRNA-34a Inhibits the Proliferation and Metastasis of Osteosarcoma Cells Both In Vitro and In Vivo

**DOI:** 10.1371/journal.pone.0033778

**Published:** 2012-03-21

**Authors:** Kang Yan, Jie Gao, Tongtao Yang, Qiong Ma, Xiuchun Qiu, Qingyu Fan, Baoan Ma

**Affiliations:** Department of Orthopedic Surgery, Tangdu Hospital, Fourth Military Medical University, Xi'an, Shaanxi, People's Republic of China; Sun Yat-sen University Medical School, China

## Abstract

**Background:**

MicroRNAs (miRNAs) are a class of endogenously expressed, small noncoding RNAs, which suppress its target mRNAs at the post-transcriptional level. Studies have demonstrated that miR-34a, which is a direct target of the p53 tumor suppressor gene, functions as a tumor suppressor and is associated with the tumor growth and metastasis of various human malignances. However, the role of miR-34a in osteosarcoma has not been totally elucidated. In the present study, the effects of miR-34a on osteosarcoma and the possible mechanism by which miR-34a affected the tumor growth and metastasis of osteosarcoma were investigated.

**Methodology/Principal Finding:**

Over-expression of miR-34a partially inhibited proliferation, migration and invasion of osteosarcoma cells in vitro, as well as the tumor growth and pulmonary metastasis of osteosarcoma cells in vivo. c-Met is a target of miR-34a, and regulates the migration and invasion of osteosarcoma cells. Osteosarcoma cells over-expressing miR-34a exhibited a significant decrease in the expression levels of c-Met mRNA and protein simultaneously. Finally, the results from bioinformatics analysis demonstrated that there were multiple putative targets of miR-34a that may be associated with the proliferation and metastasis of osteosarcoma, including factors in Wnt and Notch signaling pathways.

**Conclusion/Significance:**

The results presented in this study demonstrated that over-expression of miR-34a could inhibit the tumor growth and metastasis of osteosarcoma probably through down regulating c-Met. And there are other putative miR-34a target genes beside c-Met which could potentially be key players in the development of osteosarcoma. Since pulmonary metastases are responsible for mortality of patient carrying osteosarcoma, miR-34a may prove to be a promising gene therapeutic agent. It will be interesting to further investigate the mechanism by which miR-34a functions as a tumor suppressor gene in osteosarcoma.

## Introduction

Osteosarcoma (OS) is the most common human primary malignant bone tumor in children and young adults, which accounts for approximately 60% of malignant bone tumors in the first 2 decades of life [1]. It mainly present around regions with active bone growth and repairation, such as knee joint, lower femur and upper tibia. With a rapid expansion of our knowledge about stem cell biology, emerging evidence suggests osteosarcoma should be regarded as a kind of differentiation disease caused by genetic and epigenetic changes that interrupt osteoblast differentiation from mesenchymal stem cells. Osteosarcoma is locally destructive and has a high metastatic potential [2]. The clinical treatment for osteosarcoma is of great difficulties, and patients treated with amputation alone often died of pulmonary metastasis within one year. Thanks to the rapid development of treatment for high grade osteosarcoma which combines surgery with neoadjuvant and adjuvant chemotherapy [3], the 5-year survival rate of patients carrying osteosarcoma has been dramatically improved [4]. However, the cure rate of patients carrying osteosarcoma is still very poor and most of them eventually died of pulmonary metastases [5]. Therefore, in addition to the surgical removal of the primary tumor and the chemotherapy, the prevention of pulmonary metastases during the early stage of tumor development is also critical for the improvement of the prognosis of patients carrying osteosarcoma. Gene therapy is one such targeted technique for application to osteosarcoma and various studies have been carried out to investigate the genes that are involved in metastasis of osteosarcoma. However, the highly complex molecular mechanism of metastasis is still poorly understood. Nowadays, miRNAs have become a new research hotspot for gene therapy.

miRNAs (microRNAs) are a class of endogenous, noncoding, single stranded small regulatory RNA molecules, which are approximately 22 nucleotides in length [6]. Their coding genes, which are mainly located in cancer associated genomic regions or in fragile sites, account for approximately 1% of the entire genome [7]. miRNAs play an important role in the regulation of gene expression at the post-transcriptional level. Unlike short interfering RNAs (siRNAs), miRNAs mainly silence the expression of multiple genes instead of a single gene. It is estimated that miRNAs have the potential to regulate at least 20%–30% of all human genes [8], and that an average miRNA have more than 100 targets [9]. However, their biological function remains largely unknown and only a few mRNAs that are directly regulated by miRNAs in animals have been verified empirically. miRNAs are often deregulated in human malignancies and correlated to the regulation of many cellular processes including proliferation, differentiation, apoptosis and metastasis. miRNAs can function as either oncogenes or tumor suppressors by specifically regulating the expression of their target genes [10]. Those miRNAs whose expression is increased in tumors may be considered as oncogenes. These oncogene miRNAs usually promote tumor development by negatively regulating tumor suppressor genes. Meanwhile, some miRNAs whose expression is decreased in tumor are considered as tumor suppressor genes. Tumor suppressor miRNAs usually prevent tumor development by negatively regulating oncogenes. Recently, mounting evidence has indicated that miRNAs are attractive candidates of upstream regulators in metastatic progression, because they may regulate a number of invasion and metastasis-related genes [11], [12], [13], [14], [15], suggesting that miRNAs may be used as a potential therapeutic avenue in preventing tumor metastasis.

miR-34a is a member of an evolutionarily conserved miRNA family, miR-34s. miR-34a is a direct transcriptional target of p53 tumor suppressor [16]. The inactivating mutations of p53 often cause a decreased expression of miR-34a in tumors [17]. miR-34a functions as a tumor suppressor gene by down-regulating its targets such as CDK4, CDK6, E2F3, E2F5 et al [18], [19]. miR-34a also plays an important role in the p53-induced cell cycle arrest, cell senescence, apoptosis and other biological behavior [20]. The inactivation and absence of miR-34a is related to the pathogenesis of a variety of tumors [17], [21], [22], including osteosarcoma [23]. However, the effects of miR-34a on osteosarcoma have not been totally elucidated. Therefore, it is of great significance to further study the function and mechanism of miR-34a in Osteosarcoma.

In the present studies, we performed in vitro and in vivo experiments to evaluate the effects of miR-34a on tumor growth and metastasis of SOSP-9607 cells, as well as the expression of c-Met, because c-Met is a direct target of miR-34a and correlated to the metastasis potential of tumors. We also performed the prediction of miR-34a putative target genes which are correlated to tumor growth and metastasis by using bioinformatics analysis. For the first time, we reported that over-expression of miR-34a inhibited growth and metastasis of osteosarcoma cells both in vitro and in vivo. In addition, miR-34a could specifically down-regulate the expression of the metastasis related gene c-Met, indicating that miR-34a may function as a tumor gene suppressor through down-regulating c-Met oncogene. Finally, there are other putative miR-34a target genes beside c-Met which may mediate the miR-34a induced inhabitation of tumor growth and metastasis in osteosarcoma. We supposed that miR-34a may prove to be a promising gene therapeutic agent which functions as a tumor suppressor gene through down-regulating multiple target oncogenes in osteosarcoma.

## Results

### 1. Plasmid construction and generation of stable cells

**Figure 1:**
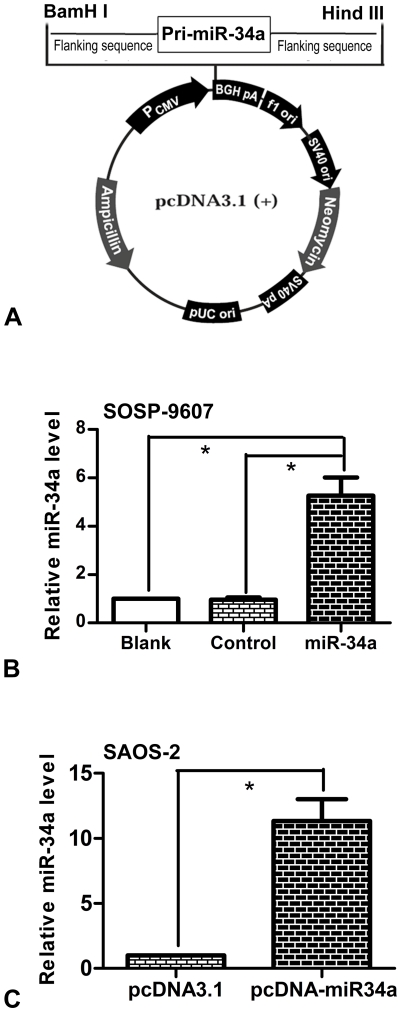
Plasmid construction and generation of stable cells. (**A**) Schematic representation of miR-34a expression vector. (**B**) Relative miR-34a expression levels in three groups of SOSP-9607 cells (Blank, SOSP-9607 cells; control, stable SOSP-9607 cells transfected with pcDNA3.1; miR-34a, stable SOSP-9607 cells transfected with pcDNA-miR34a.). (**C**) Relative miR-34a expression levels in SAOS-2 cells transiently transfected with pcDNA3.1 and pcDNA-miR34a respectively. U24 small nucleolar RNA (RNU24) was used as an internal loading control to normalize the results. Data are presented as means±SD. *P<0.01 (n = 3) is considered as statistically significant.

To facilitate the investigation of the effects of miR-34a on osteosarcoma, a has-miR-34a eukaryotic expression vector, named pcDNA-miR34a, was constructed (Figure 1A). The constructs was then verified by DNA sequencing (Figure S1). SOSP-9607 cells were transfected with either pcDNA-miR34a or pcDNA3.1 and then G418-slected for 6 weeks to generate two stable SOSP-9607 cells (stable SOSP-9607 cells transfected with pcDNA3.1 and pcDNA-miR34a, respectively). Therefore, experiments with SOSP-9607 cells were divided into three groups as blank group (SOSP-9607 cells), control group (stable SOSP-9607 cells transfected with pcDNA3.1) and miR-34a group (stable SOSP-9607 cells transfected with pcDNA-miR34a).

The miR-34a expression levels in three groups were measured using Stem-loop Real-time RT-PCR. The results showed that cells in miR-34a group expressed a higher level of miR-34a as compared with control group and blank group,respectively. However, we did not find significant difference between control group and blank group (Figure 1B). The miR-34a expression levels in SAOS-2 cells transiently transfected with either pcDNA3.1 or pcDNA-miR34a was also measured, and a similar result was shown (Figure 1C). These results indicated that pcDNA-miR34a can up-regulate miR-34a expression in both SOSP-9607 cells and SAOS-2 cells, which facilitated the further study of miR-34a functions in osteosarcoma.

### 2. miR-34a inhibits proliferation of osteosarcoma in vitro

**Figure 2:**
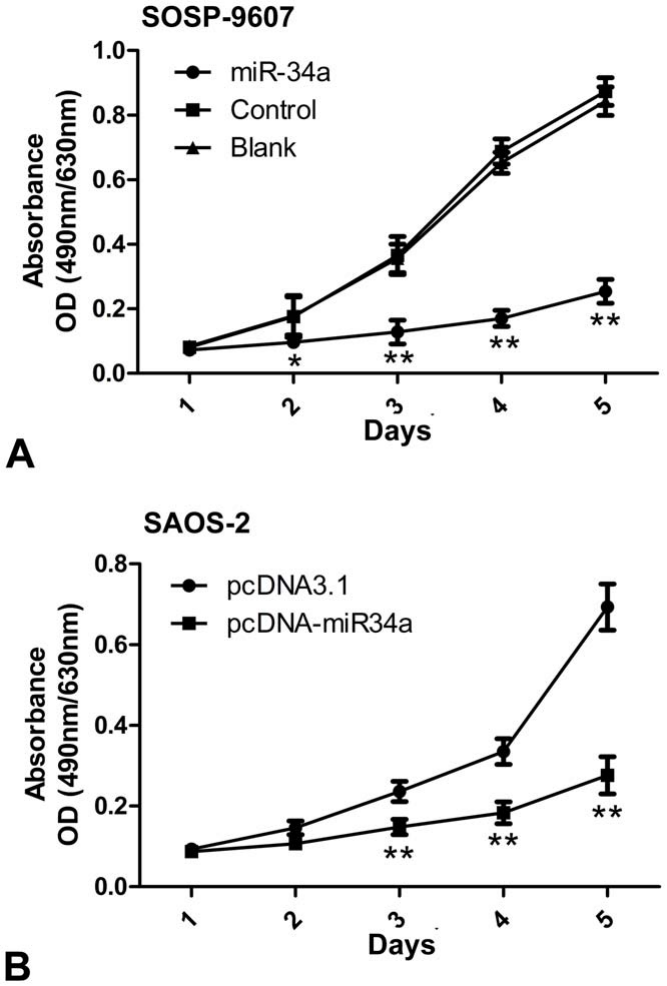
miR-34a inhibits proliferation of osteosarcoma in vitro. Every 24 h, MTT assay was performed on three groups of SOSP-9607 cells (Blank, SOSP-9607 cells; control, stable SOSP-9607 cells transfected with pcDNA3.1; miR-34a, stable SOSP-9607 cells transfected with pcDNA-miR34a) and SAOS-2 cells (pcDNA3.1, SAOS-2 cells transiently transfected with pcDNA3.1; pcDNA-miR34a, SAOS-2 cells transiently transfected with pcDNA-miR34a) respectively (**A, B**). The viable cell number was evaluated as the value of the absorbance at 490 nm with a reference wavelength of 630 nm. Values of optical density (OD) are expressed as means±SD. *P<0.05, ** p<0.01 (n = 3) are considered as accepted as statistically significant.

To investigate the effects of miR-34a on the proliferation of osteosarcoma cells, MTT assay was performed following the procedure described in Methods every 24 h. And then proliferation curve was depicted. The results demonstrated that cells in miR-34a group exhibited significant declines in proliferation capacity as compared with cells in control group and blank group, which exhibits a negative relationship with the exogenous miR-34a level (Figure 2A). We also tested SAOS-2 cells transiently transfected with either pcDNA3.1 or pcDNA-miR34a (Figure 2B) and SOSP-9607 cells transiently transfected with either miR-34a mimics or inhibitors (Figure S2A, B). The results were similar to that of stable transfected SOSP-9607 cells.

### 3. miR-34a inhibits migration and invasion of osteosarcoma in vitro

**Figure 3:**
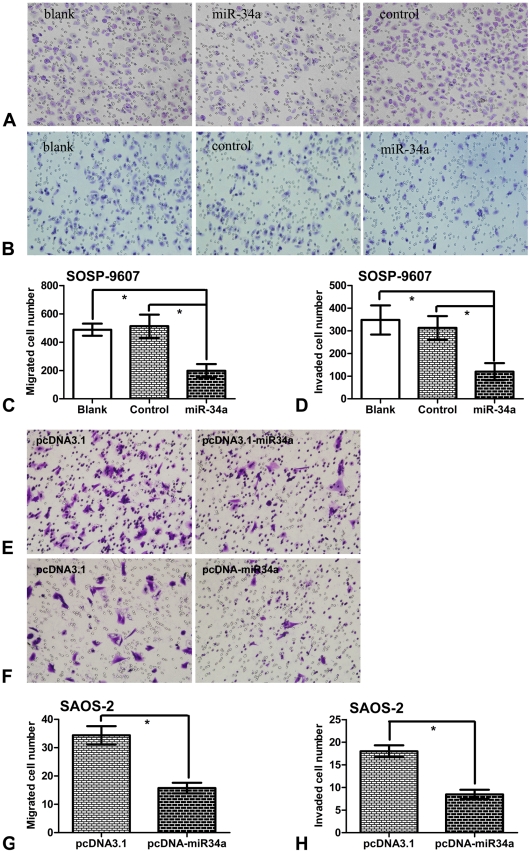
miR-34a inhibits migration and invasion of osteosarcoma in vitro. Representative photographs of migrated and invaded SOSP-9607 cells (Blank, SOSP-9607 cells; control, stable SOSP-9607 cells transfected with pcDNA3.1; miR-34a, stable SOSP-9607 cells transfected with pcDNA-miR34a) on the membrane at a magnification of 100× (**A, B**). Quantitative results for the migration and invasion ability of each group of SOSP-9607 cells were shown as migrated and invaded cell number, 16 h after incubation (**C, D**). Representative photographs of migrated and invaded SAOS-2 cells (pcDNA3.1, SAOS-2 cells transiently transfected with pcDNA3.1; pcDNA-miR34a, SAOS-2 cells transiently transfected with pcDNA-miR34a) on the membrane at a magnification of 100× (**E, F**). Quantitative results for the migration and invasion ability of SAOS-2 cells were shown as migrated and invaded cell number, 16 h after incubation (**G, H**). Data are expressed as means±SD. *P<0.01 (n = 3) is considered as statistically significant.

It has been reported that hepatocellular carcinoma cells transfected with miR-34a mimics are inhibited of both migration and invasion [24], and the expression of miR-34a is associated with the tumorigenesis of osteosacoma [23]. However, there is no study on the role of miR-34 in osteosarcoma metastasis. Therefore, in this part, we performed transwell migration and invasion assay to investigate the effects of miR-34a on the migratory and invasive behaviors of osteosarcoma cells in vitro. The results demonstrated that cells in miR-34a group exhibited significant declines in migration and invasion capacities as compared with cells in control group and blank group respectively (Figure 3A, B, C, D). However, there is no significant difference between blank group and control group. We also tested SAOS-2 cells transiently transfected with either pcDNA3.1 or pcDNA-miR34a (Figure 3E, F, G, H) and SOSP-9607 cells transiently transfected with either miR-34a mimics or inhibitors (Figure 2C, D, E, F), the results were similar to that of stable transfected SOSP-9607 cells. , strongly indicated that miR-34a was an important participant in the reduction of migratory and invasive potential of osteosarcoma in vitro.

### 4. miR-34a inhibits tumor growth of osteosarcoma in vivo

Given these findings in vitro, animal studies were conducted to further evaluate the effect of miR-34a on orthotopic tumor growth in athymic nude mice. Two groups of stable transfected cells (control group, stable SOSP-9607 cells transfected with pcDNA3.1; and miR-34a group, stable SOSP-9607 cells transfected with pcDNA-miR34a) were injected into proximal tibia of young nude mice as described in methods, respectively.

**Figure 4:**
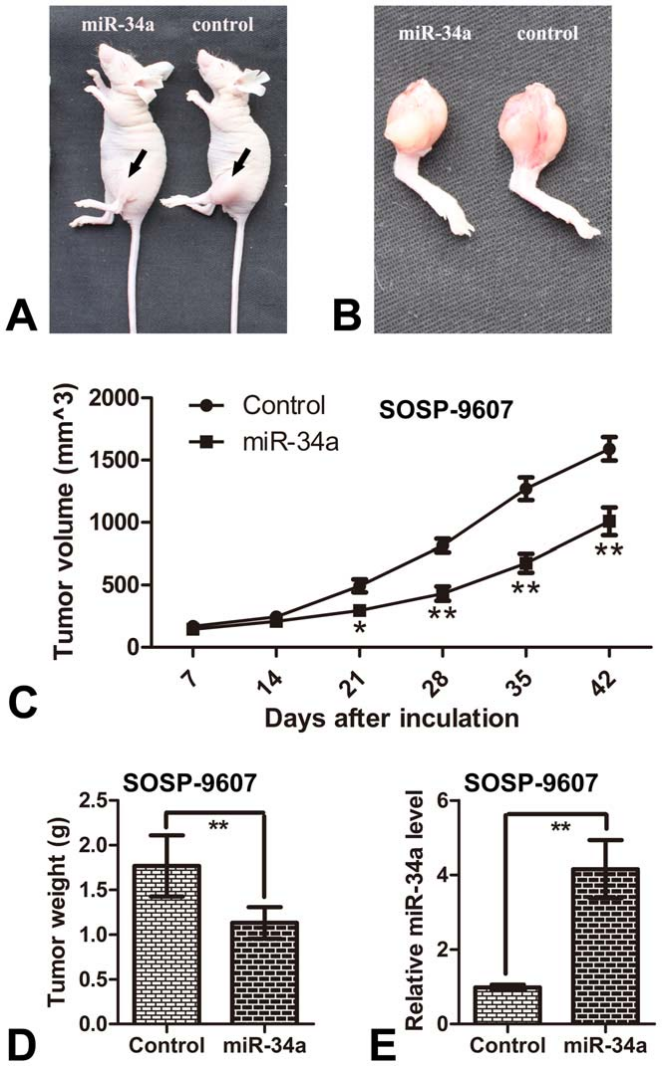
miR-34a inhibits tumor growth of osteosarcoma in vivo. The results showed that the growth velocities of orthotopic tumors in miR-34a group (stable SOSP-9607 cells transfected with pcDNA-miR34a.) decreased as compared with control group (stable SOSP-9607 cells transfected with pcDNA3.1). (**A**) Representative photographs of tumors (arrows) on the left legs of mouse. (**B**) Representative photographs of orthotopic tumors harvested 42 days after inoculation. (**C**) Tumor growth curves measured after the inoculation. The length (L) and width (W) of tumor measured every 7 days after inoculation, and the volume of tumor was calculated according to the formula: volume = 1/2×L×W^2^. (**D**) Orthotopic tumor weights 42 days after inoculation. Data are presented as means±SD. (**E**) 42 days after inoculation, miR-34a expression levels in Orthotopic tumors were tested and showed in relative miR-34a levels. *P<0.05, ** P<0.01 (n = 6) are considered as statistically significant.

To evaluated tumor growth, the length (L) and width (W) of orthotopic tumor were measured every 7 days post inoculation. The volume of tumor was calculated according to the formula: volume = 1/2×L×W^2^, and the growth curve of orthotopic tumor was depicted. The results demonstrated that cells in control group formed progressively growing solid tumors in all mice. By contrast, cells in miR-34a group produced much smaller tumors (Figure 4A, C). 42 days after inoculation, the mice were sacrificed and the orthotopic tumors were harvested and weighed. The mean tumor weight±SD of orthotopic tumors were as follows: miR-34a group 1.132±0.177 g, control group 1.768±0.341 g (Figure 4B, D). Meanwhile, the miR-34a expression levels in the orthotopic tumors were also tested, and the result showed that the orthotopic tumors in the miR-34a group expressed higher miR-34a levels as compared with control group (Figure 4E). Both of the results indicated that ectogenous miR-34a can significantly inhibit the tumor growth of osteosarcoma in vivo.

### 5. miR-34a inhibits pulmonary metastasis of osteosarcoma in vivo

**Figure 5:**
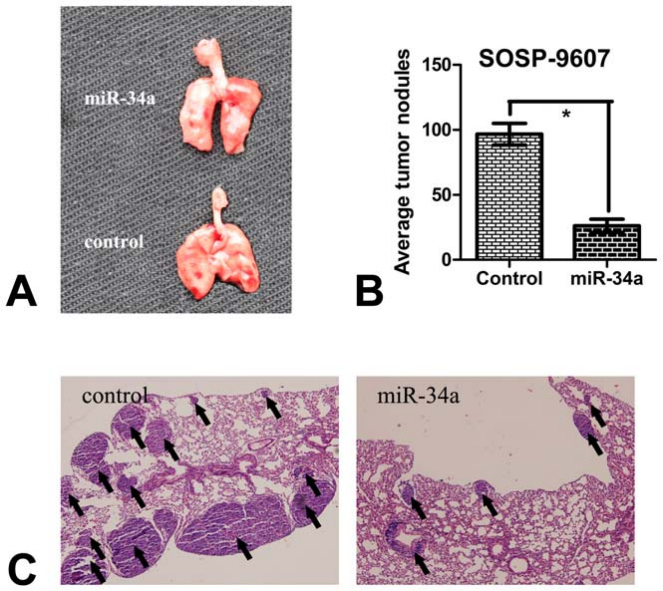
miR-34a inhibits pulmonary metastasis of osteosarcoma in vivo. (**A**) Representative macroscopic pictures of mouse lungs, 42 days after inoculation. (**B**) Graph displaying the total number of tumor nodules per lung in control group (stable SOSP-9607 cells transfected with pcDNA3.1) and miR-34a group (stable SOSP-9607 cells transfected with pcDNA-miR34a.). Data are presented as means±SD. *P<0.01 (n = 6) is considered as statistically significant. (**C**) Representative photographs of H&E stained spontaneous lung metastases. The results showed that most of the tumor nodules were organized in a predominantly peripheral distribution in both groups. However, tumor nodules (arrows) in miR-34a group were smaller and distributed in a lower concentration as comparing with control group.

To evaluate the pulmonary metastasis potential of cells in miR-34a group and control group, 42 days after inoculation, the mouse lungs in both groups were harvest and the tumor nodules on the surface of the lung were counted and photographed (Figure 5A). An average of 26.2±12.4 metastatic tumor nodules were detected per lung in miR-34a group, while mice in control group produced an average of 96.7±20.5 metastatic tumor nodules per lung (Figure 5B), indicating that miR-34a significantly decreased tumor colonization in the lung.

Histological examination of the lung sections showed that most of the tumor nodules were organized in a predominantly peripheral distribution in both groups. However,there was a significant difference in tumor nodules concentration and size in lung histology sections. The lungs of miR-34a group mice contained less and smaller spontaneous metastases as comparing with control group (Figure 5C). Taken together, these results strongly suggest that miR-34a can inhibit osteosarcoma metastasis and might be prevention of metastasis and recurrence in osteosarcoma patients.

### 6. c-Met is a target of miR-34a, and regulates the migration and invasion of osteosarcoma cells

**Figure 6:**
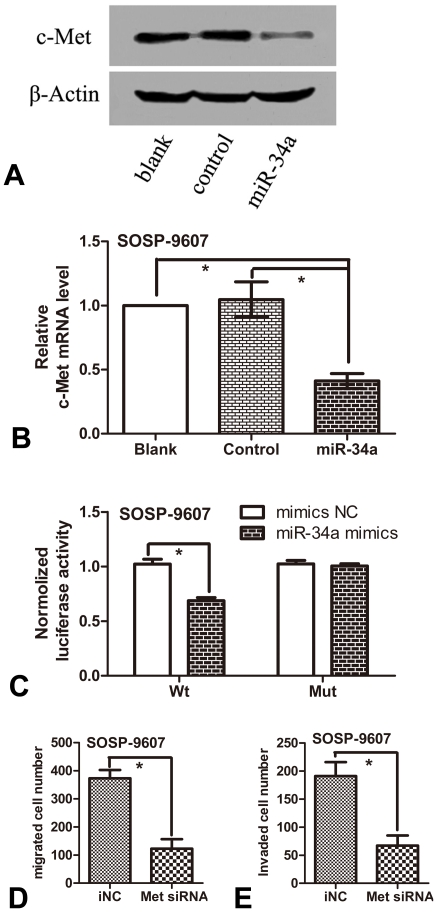
c-Met is a target of miR-34a, and regulates the migration and invasion of osteosarcoma cells. (**A**) Western blotting analysis of c-Met protein expression. (**B**) qRT-PCR analysis of c-Met mRNA expression. The mRNA levels of c-Met in three groups of SOSP-9607 cells (blank, SOSP-9607 cells; control, stable SOSP-9607 cells transfected with pcDNA3.1; miR-34a, stable SOSP-9607 cells transfected with pcDNA-miR34a.) were normalized against that of glyceraldehyde-3-phosphate dehydrogenase (GAPDH) which served as an internal control. (**C**) The result from luciferase assay showed that the luciferase activity of the pmiR-Met UTR-Wt construct was significantly inhibited after the introduction of miR-34a mimics. Meanwhile, mutations of the two c-Met 3′UTR-binding sites abolished the ability of miR-34a to regulate luciferase expression. (**D, E**) SOSP-9607 cells were transiently transfected with 100 nM of c-Met siRNA or inhibitor NC (Genepharma, China), respectively. 48 h later, the migration and invasion assay were performed. Quantitative results for the effects of c-Met siRNA on the migration and invasion ability of SOSP-9607 cells were shown as migrated and invaded cell number. The results are presented as means±SD. *P<0.01 (n = 3) is accepted as statistically significant.

Studies have reported that miR-34a directly repressed the expression of c-Met in HeLa cells [16], suppressed brain tumor growth by targeting c-Met [25], and acted as a tumor suppressor in uveal melanoma cell proliferation and migration through the down-regulation of c-Met [26]. However, considering that the miR-34a-c-Met pathway may show different patterns in different cell backgrounds, we further investigated whether over-expression of miR-34a also down-regulated the expression of c-Met in osteosarcoma cells. We detected the mRNA level of c-Met by RT-PCR and the -protein levels of c-Met by western blotting in three groups of stable SOSP-9607 cells. The results from Western blotting demonstrated that the endogenous c-Met protein level in miR-34a group cells exhibited remarkable decrease as compared with blank and control groups respectively (Figure 6A). The results from RT-PCR demonstrated that the endogenous c-Met mRNA level in miR-34a group cells was also significantly decreased (Figure 6B). These results together indicate that miR-34a down-regulates c-Met expression in osteosarcoma at the translational level and reduces mRNA stability simultaneously.

We also repeated the luciferase assay to validate c-met as a target of miR-34a in osteosarcoma cells SOSP-9607, and tested whether c-Met inhibits the migration and invasion of SOSP-9607 cells as that in other cells. The results demonstrated that, in SOSP-9607 cells, the luciferase activity of the pmiR-Met UTR-Wt construct was significantly inhibited after the introduction of miR-34a, similar to those reported by others [27]. Meanwhile, mutations of the two c-Met 3′UTR-binding sites abolished the ability of miR-34a to regulate luciferase expression (Figure 6C). And as expected, c-Met siRNA significantly inhibited the migration and invasion of SOSP-9607 -cells (Figure 6D, E).

### 7. Putative Targets of miR-34a

Bioinformatics methods based on sequence similarity between miRNAs and mRNAs were used to predict the putative target genes of miR-34a. We predicted putative miR-34a target genes by using online softwares, such as TargetScan 5.1 and PicTar, and finally obtained several putative target genes that are correlated with the tumor growth or metastasis (Table 1, 2).

**Table 1:** Putative Targets of miR-34a.

Putative targets	Software	Fuctions and Pathways
BCL6	pictar	proliferation; apoptosis.
CCNE2	pictar	cell cycle; metastasis.
CD97	targetscan	cell motility; cell adhesion.
CSNK1G3	pictar	Wnt signaling pathway.
CTNND1	pictar	cell adhesion; Wnt signaling pathway.
DLL1	pictar	cell adhesion; Notch signaling pathway.
DKK1	miRanda	Wnt signaling pathway.
GAS1	targetscan	apoptosis; cell cycle arrest; proliferation.
IGFBP3	pictar	apoptosis; proliferation.
LEF1	pictar	Wnt signaling pathway.
PGEA1	miRanda	Wnt signaling pathway.
POFUT1	pictar	angiogenesis; Notch signaling pathway.
R-RAS	pictar	cell adhesion; cell migration.
RUNX2	TargetScan	cell proliferation; cell migration.
UHRF2	Pictar	cell cycle; proliferation.
VCL	pictar	Cell migration; cell adhesion.
VEGFA	Targetscan	Angiogenesis.

**Table 2:** Experimentally identified Targets of miR-34a.

Experimental identified targets	Software	Functions and pathways
BCL2	pictar	proliferation; apoptosis.
CCND1	pictar	cell cycle arrest; proliferation; cell migration; Notch signaling pathway; Wnt signaling pathway.
CDK6	pictar	cell cycle arrest; Proliferation.
E2F3 [38] [40]	pictar	cell cycle arrest; proliferation.
JAG1	pictar	Proliferation; Cell Invasion; Notch signaling pathway.
Mek1	pictar	cell motility; proliferation.
MET	pictar	Proliferation; cell invasion.
MYCN	miRanda	Apoptosis; cell cycle; proliferation.
NOTCH1	Pictar	Proliferation; cell invasion; Notch signaling pathway.
NOTCH2	pictar	Proliferation; Notch signaling pathway.
SIRT1	pictar	Cell cycle arrest; Apoptosis; tumor growth; Notch signaling pathway.
WNT1	pictar	Proliferation; cell migration; Wnt signaling pathway

## Discussion

A large body of evidence has indicated that miRNAs are frequently deregulated in a variety of human malignancies [28]. Studies showed a direct link between miRNA function and oncogenesis which is supported by examining the expression of miRNAs in clinical samples [29], [30]. The profiling of miRNA expression showed that most of them are down-regulated in tumors compared to normal tissues [31], like let-7 in lung cancers [32] and miR-127 in human bladder cancers [33]. However, there are other miRNAs which are up-regulated in tumors, like miR-150 in gastric cancer [34], miR-21 in prostate cancer [35] and miR-17-92 cluster in renal cell carcinoma [36]. We focused on miR-34a because not only previous reports demonstrated that the expression of miR-34a was significantly decreased in primary osteosarcoma samples as compared with adjacent normal tissues [23], but also the mutations of p53 tumor suppressor gene, which directly regulates the expression of miR-34a, was also found in 20–60% of sporadic osteosarcomas [16], [37]. Both of the previous reports suggested that miR-34a may function as a tumor suppressor in osteosarcoma.

miR-34a is a member of an evolutionarily conserved miRNA family, miR-34s. Initial links to tumorigenesis emerged from Welch et al. who found that miR-34a, whose encoding gene is on chromosome 1p36, is correlated with tumor occurrence and frequently missed in human neuroblastoma [38]. miR-34 family are direct transcriptional targets of p53 tumor suppressor [16]. There is a highly conserved p53 binding site which is approximately 30 kb above the miR-34a encoding gene [39]. The inactivating mutations of p53 often cause a decreased expression of miR-34a in tumors [17]. miR-34a plays its tumor inhibitory effect by down-regulating its targets such as CDK4, CDK6, E2F3, E2F5 et al [18], [19]. miR-34a also plays an important role in the p53-induced cell cycle arrest, cell senescence, apoptosis and other biological behavior [20]. The inactivation and absence of miR-34a is related to the pathogenesis of a variety of tumors [17], [21], [22], including osteosarcoma [23]. Moreover, Li et al. found that miR-34a inhibits migration and invasion of human hepatocellular carcinoma cells [24]. Pang et al. found that MiR-34a suppresses invasion of cervical carcinoma and choriocarcinoma cells [40]. However, the functions of miR-34a in osteosarcoma have not been totally elucidated.

Uncontrolled cell proliferation and aggressive tumor cell metastasis are two essential steps during cancer progression. The former lead to the orthotopic tumor growth, and the latter promote tumor transfering to distant sites. Therefore, in this study we investigated the effects of miR-34a on tumor growth and metastasis of osteosarcoma. we demonstrated that over-expression of miR-34a significantly suppresses proliferation,migration and invasion of SOSP-9607 cells in vitro. These results indicate that miR-34a plays an important role in the development of osteosarcoma. However, we can't imprudently assume that miR-34a would also have biological activity in vivo. Therefore, we chose a mouse model to further investigate effects of miR-34a on tumor growth and pulmonary metastasis. The results demonstrated that miR-34a also significantly inhibited the capacities of orthotopic tumor growth and lung metastasis in vivo. Because we couldn't maintain the G418 selection in vivo, the selected cells may not maintain miR-34a over-expression as that in vitro. So, we further tested the miR-34a expression levels in the orthotopic tumors 6 weeks after inoculation. The result showed that the miR-34a expression level of G418 sellected cells, in the orthotopic tumors after 6 weeks of inoculation, was indeed over-expressed as that in vitro. Therefore, up-regulated expression of miR-34a was very effective on inhibiting the tumor growth and metastasis behaviors of osteosarcoma cells both in vitro and in vivo. These results indicate that miR-34a functions as a tumor suppressor gene and can be used as a potential target in the gene therapy of osteosarcoma.

Metastatic models of tumorigenesis are crucial to understand the Metastatic behavior of cancer. However, the Metastatic models of osteosarcoma in experimental animals are rare. Usually, the metastatic models were conducted in nude mouse by injecting human osteosarcoma cells either intravenously or subcutaneously [41]. However, these models are not clinically relevant because osteosarcoma cells are not spontaneously arisen and will not grow in the site with proper microenvironment [42]. In this study, we choose a spontaneous metastatic model, in which orthotopic ransplantation of osteosacoma cells were performed and then spontaneous pulmonary metastases were observed [43], [44]. The tumor progression and metastases development follow the clinical course of osteosarcoma in this animal model, because the micro-environment of nude mice tibia is similar to the actual situation. Moreover, with this model, we can study the properties of orthotopic tumor growth and spontaneous pulmonary metastasis of osteosarcoma cells simultaneously.

The Receptor tyrosine kinase (RTK) c-Met is a cell surface receptor for hepatocyte growth factor (HGF) [45]. The Met oncogene is up-regulated in a variety of tumor cells similar in scope to p53 mutants [46], [47]. HGF-mediated activation of c-Met results in a complex genetic program referred to as “invasive growth”, including proliferation, invasion, and angiogenesis [48], [49]. In tumor cells, c-Met activation triggers a diverse series of signaling cascades resulting in cell growth, proliferation, invasion, and protection from apoptosis [50]. Studies have shown that miR-34a suppresses brain tumor growth by targeting c-Met and Notch [25], suppresses proliferation and migration through the down-regulation of c-Met in uveal melanoma cells [26], and inhibits migration and invasion through down-regulation of c-Met expression in human hepatocellular carcinoma cells [24]. The activities associated with Met signalling including migration, invasion and colony formation in soft agar were blocked by a small molecule Met inhibitor [51]. Therefore, in SOSP-9607 cells, we further investigated whether over-expression of miR-34a also down-regulated the expression of c-Met and whether c-Met inhibited the migration and invasion as that in other cells. We examined the c-Met expression level of osteosarcoma cells in three groups SOSP-9607 cells, and observed a significant decrease of c-Met mRNA and protein levels in miR-34a group cells as compared with blank group and control group. As expected, c-Met also regulated the migration and invasion of SOSP-9607 cells, and c-Met is indeed a direct target of miR-34a in SOSP-9607 cells. These results indicated that miR-34a may suppress tumor growth and metastasis in osteosarcoma cells through down-regulating c-Met oncogene. It is supposed that the miR-34a-c-Met pathway might be a general regulator of tumor growth and metastasis in a wide range of human malignances, including brain tumor, uveal melanoma, hepatocellular carcinoma and osteosarcoma.

Unlike siRNAs which silence the expression of a single gene, miRNAs mainly silence the expression of multiple genes simultaneously. It is estimated that an average miRNA have more than 100 targets [9], and miRNAs have the potential to regulate at least 20%–30% of all human genes [8]. It is likely that miR-34a may also regulate other genes beside c-Met. Therefore, to thoroughly understand the regulatory mechanism of miR-34a in osteosarcoma,it is critical to identify more target genes that mediate the miR-34a induced regulation of tumor growth and metastasis.

Predicting and identifying the miR-34a-targeting genes offer experimental basis for further research on regulatory mechanism of miR-34a. By using TargetScan 5.1 and PicTar, we predicted putative genes of miR-34a which have not been experimentally identified yet, and finally obtained several putative targets which are correlated with tumor growth or metastasis, such as BCL6, CCNE2, CD97, CSNK1G3, CTNND1, DLL1, DKK1, GAS1, IGFBP3, LEF1, PGEA1, POFUT1, R-RAS, RUNX2, UHRF2, VCL, VEGFA, etc. (Table 1).

And then, we reviewed experimentally identified miR-34a target genes, such as BCL2 [52], CCND1 [18],CDK6 [18], E2F3 [38], JAG1 [40], Mek1 [53], MET [25], MYCN [54], NOTCH1 [55], NOTCH2 [55], SIRT1 [56], WNT1 [57] et al (Table 2). Among them, there are several genes, which mediate the inhibition of miR-34a induced metastasis in human malignances, such as Notch1 and JAG1 in cervical carcinoma and choriocarcinoma cells [40] and c-Met in in human hepatocellular carcinoma cells [24]. Meanwhile, there are several genes which mediate the miR-34a induced tumor growth inhibition, such as BCL2, E2F3 and MYCN in neuroblastoma [52], [54], mitogen-activated protein kinase kinase 1 (MEK1) in human chronic myelocytic leukemia cell line K562 [53] and SIRT1 in prostate cancer PC3 cells [58].

The dysregulation of several evolutionarily conserved signaling pathways in osteosarcoma tumor samples and cell lines have been repeatedly found. These signaling pathways include the Hedgehog (Hh), TGF-β/BMP, ERBB, Notch and Wnt signaling pathways. Notch signaling pathway participates in a variety of cellular processes, including cell fate specification, differentiation, proliferation, apoptosis, adhesion, epithelial-mesenchymal transition, migration, and angiogenesis. Activation of Notch signaling contributes to the pathogenesis of human osteosarcomas andthe inhibition of the Notch signaling may be a therapeutic approach for the treatment of osteosarcoma [59]. Wnt signaling pathway plays an important role in regulating cell proliferation and differentiation. Deregulation of Wnt signaling pathway has been implicated in many human diseases, ranging from cancers to skeletal disorders [60]. Dickkopf 3 inhibits invasion and motility of osteosarcoma cells SAOS-2 by modulating the Wnt-beta-catenin pathway [61]. Interestingly, many genes in Notch and Wnt signaling pathways are putative targets of miR-34a. For instance, CCND1, CSNK1G3, CTNND1, DKK1, LEF1, PGEA1 and WNT1 are members of Wnt signaling pathway, while CCND1, DLL1, JAG1, NOTCH1, NOTCH2, POFUT1 and SIRT1 are members of Notch signaling pathway (Table 1, 2). Therefore, we inferred that miR-34a may play an important role in inhibiting tumor metastasis and proliferation through down-regulating multiple target genes, including genes in Notch and Wnt signaling pathways.

In conclusion, the results presented here demonstrated that miR-34a has great biological effects on the growth and metastasis of osteosarcoma cells both in vitro and in vivo. Over-expression of miR-34a down-regulated the expression of c-Met protein and mRNA simultaneously, suggesting that miR-34a functions as tumor suppressors probably through down-regulating c-Met in osteosarcoma. Furthermore, there are other putative miR-34a target genes which could potentially be key players in the growth and metastasis of osteosarcoma cells. However, these putative targets should be further verified to provide conclusive evidence. Finally, because pulmonary metastases are responsible for mortality of patient carrying osteosarcoma, miR-34a may prove to be a promising gene therapeutic agent. It will be interesting to verify the putative target genes and further investigate the mechanism by which miR-34a functions as a tumor suppressor gene in osteosarcoma.

## Materials and Methods

### Ethics statement

All the animal operations were performed under the rules provided by Declaration of Helsinki and approved by the Animal and Ethics Review Committee, Fourth Military Medical University, Xi' an, Shaanxi, China (approval ID:2009043). The anesthesia method and orthotopic transplantation procedures were based on the methods reported previously [62], [63].

### Cell culture

Human osteosarcoma cells SOSP-9607 and SAOS-2 were established and reserved in our laboratory as described previously [43], [64]. SOSP-9607 cells was maintained in RPMI 1640 medium (HyClone, USA) supplemented with 10% fetal bovine serum (FBS) (HyClone, USA), 2.0 mM L-glutamine, 100 U/ml penicillin, and 100 ug/ml streptomycin, and incubated at 37°C in humidified incubator supplemented with 5% CO2 and 95% air. SAOS-2 cells were maintained in the same conditions, except that DMEM medium was used.

### Plasmid construction and generation of stable cells

The procedure of miR-34a eukaryotic expression vector construction was referred to the method described previously [65]. In brief, an approximately 389 base pairs (bp) DNA fragment, which covering the pri-miR-34a (HGNC:31635; MIM:611172; miRBase:MI0000268) and native flanking sequence, were PCR-amplified from SOSP-9607 genomic DNA using the following primers: Pri-miR34a FP/BamHI, 5′- CGG GAT CCC CTC CTG CAT CCT TTC TTT -3′; Pri-miR34a RP/HindIII, 5′- CGG AAT TCC CTG TGC CTT TTT CCT TCC -3′. The correct sequences of amplified fragment were verified by sequencing, double digested with HindIII and BamHI, and cloned into pcDNA3.1 vector (Invitrogen), carrying neomycin resistance gene. The constructs was then verified by DNA sequencing.

Transfection was performed using the Lipofectamine™ 2000 transfection reagent (invitrogen) according to the manufacturer's instructions. For establishing stable transfectants, SOSP-9607 cells were transfected with either pcDNA-miR34a vector or pcDNA3.1 vector. 24 h after transfection, the tansfected cells were selected for 6 weeks in the presence of G418 at a concentration of 400 ug/ml. The stable transfectants were then expanded and the expression of miR-34a was evaluated by real time RT-PCR.

### Reverse transcription and quantitative real-time PCR

Total RNA containing miRNA and mRNA was extracted from cells with Trizol Reagent (Invitrogen), or from paraformalin-fixed, paraffin-embedded (FFPE) tissues with RecoverAll™ Total Nucleic Acid Isolation Kit (Ambion, Catalog Number: AM1975), according to the manufacturer's instructions. All RNA extractions were carried out in designated sterile laminar flow hood using RNase/DNase-free laboratory ware. The integrity and purity of total RNA was verified by UV spectrophotometry and gel-electrophoresis on formaldehyde denaturation gel. RNA extraction, and qRT assay were performed in separate designated rooms to prevent cross-contamination.

For evaluating the miR-34a expressing levels, quantification using the TaqMan microRNA assays was performed using two-step RT-PCR according to the manufacturer's instructions. In the reverse transcription (RT) step, cDNA was reverse transcribed from total RNA sample using specific miR-34a primers from the Taqman MicroRNA Assays kit (Applied Biosystems, Product ID: 000426) and reagents from the TaqMan MicroRNA Reverse Transcription Kit (Applied Biosystems, Part Number: 4366596). In the Polymerase Chain Reaction (PCR) step, PCR products were amplified from cDNA samples using the Taqman MicroRNA Assays kit together with the TaqMan Universal PCR Master Mix (Applied Biosystems, Part Number: 4304449). The real-time PCR results were normalized against an internal control U24 small nucleolar RNA (RNU24), and then expressed as fold changes.

For evaluating the c-Met expressing levels, 1 ug of total RNA was used for reverse transcription with iScript cDNA Synthesis Kit (Bio-Rad, USA) according to the manufacturer's instructions. Quantitative RT-PCR was performed with iQ SYBR Green Supermix (Bio-Rad,USA) according to the manufacturer's instructions on Real-time PCR Instrument ABI-PRISM 7000 (Applied Biosystems, CA). The sequences of the forward and reverse primers for c-Met were 5′- ACT CCC CCT GAA AAC CAA AGC C -3′ and 5′- GGC TTA CAC TTC GGG CAC TTA C -3′. The sequences of the forward and reverse primers for glyceraldehyde-3-phosphate dehydrogenase (GAPDH) were 5′- AGC CAC ATC GCT CAG ACA -3′ and 5′- GCC CAA TAC GAC CAA ATC C -3′. The expression level GAPDH was used as an internal control to normalize the amount of cDNA used for each PCR reaction. The real-time PCR results were presented as c-Met mRNA intensity/GAPDH mRNA intensity and then expressed as fold changes.

### Cell proliferation assay by methyl thiazole tetrazolium

Cells proliferation capacity was evaluated with an MTT assay, which was performed following standard procedure in 96-well plates. In brief, cells were seeded at a density of 2,000 cells per well containing 100 ul of culture medium and cultured overnight. Every 24 h interval, 20 ul of 5 mg/ml MTT(Dimethyl thiazolyl diphenyl tetrazolium, Sigma)reagent was added to each well and cells were further incubated for 4 h at 37°C. Then medium was removed, and 100 ul of DMSO (dimethyl sulfoxide) was added to each well to dissolve the formazan. The optical density (OD) was evaluated by measuring the absorbance, with a test wavelength of 490 nm and a reference wavelength of 630 nm. Wells without cells (DMSO alone) were used as blanks. There were 6 wells in each group, the experiments were repeated three times independently and the results were given as means±SD.

### Migration and invasion assay

The invasive potential of cells was measured in 6.5 mm Transwell with 8.0 µm Pore Polycarbonate Membrane Insert (cat. 3422, Corning, NY) according to the manufacturer's instructions. The filter of top chamber was matrigel-coated with 50 ul of diluted matrigel following the standard procedure and incubated at 37°C for 2 h. The lower chambers were filled with 600 ul of RPMI medium 1640 containing 5% fetal bovine serum (FBS) as chemoattractant. Cells were serum-free-starved overnight, and then harvested and resuspended in migration medium (RPMI-1640 medium with 0.5% BSA). Then the suspension of 5,000 cells in 100 ul migration medium was added into each top chamber. After the cells were incubated for 16 h, the non-invading cells that remained on the upper surface were removed with a cotton swab. The invasive cells on the lower surface of the membrane insert were fixed with 4% paraformaldehyde for 30 min, permeabilized with 0.2% Triton X-100 at room temperature for 15 min, and then stained with 0.1% crystal violet for 5 min. The number of cells on the lower surface, which had invaded through the membrane, was counted under a light microscope in five random fields at a magnification of 100×. The experiments were repeated three times independently and results were given as means±SD.

The procedure for transwell migration assays were the same as the transwell invasion assay except that the filter of top chamber was not coated with matrigel.

### Animals and operative procedure

Four-week-old female nude mice (BALB/c, nu/nu; animal centre of the Fourth Military Medical University in China (FMMU)), 17.0–22.0 g in weight,were maintained under specific pathogen-free conditions with 12-h light/12-h dark cycle at 26–28°C and 50–65% humidity. Animal feed and underpad, which were purchased from Experimental Animal Center of Fourth Military Medical University, were autoclaved and vacuum packed. The water was adjusted to the PH-value of 2.8 and autoclaved before use.

Animal experiment was performed to evaluate orthotopic tumor growth and spontaneous pulmonary metastasis properties of osteosarcoma cells in vivo. In brief, two groups SOSP-9607 cells (control group, stable SOSP-9607 cells transfected with pcDNA3.1; miR-34a group, stable SOSP-9607 cells transfected with pcDNA-miR34a) were harvested by treatment with trypsin-EDTA (Invitrogen), washed twice with PBS, and resuspended in PBS. Then osteosarcoma cells suspension of 100,000 cells in 100 ul were injected into the proximal tibia of each anesthetized nude mice (n = 6 animals per group). Every 7 days post inoculation, the length and width of individual orthotopic tumor from each mouse were measured with calipers, and the volume (mm^3^) of orthotopic tumor was calculated according to the formula: 1/2× length × width^2^ [66], where length is the longer diameter, and width the shorter one. The curve of orthotopic tumor growth was depicted. 42 days after inoculation, mouse lungs and orthotopic tumors were harvested. Then the orthotopic tumors were weighed, the miR-34a expression levels in the orthotopic tumors were tested by real time RT-PCR, and the number of pulmonary matastatic tumor nodules was counted under a low-powered dissecting stereomicroscope. Finally, mouse lungs were fixed with 10% neutral-buffered formalin, embedded in paraffin, sectioned at 6 um and stained with H&E (hematoxylin and eosin). The pulmonary metastases were imaged under a light microscope at a magnification of 100×.

### Protein extraction and western blotting analysis

Total proteins were extracted from the cells using RIPA buffer with 0.5% sodium dodecyl sulfate (SDS) in the presence of 3% proteinase inhibitor cocktail (Sigma, St. Louis, MO, USA). After being lysed on ice for 30 min, the lysate was centrifuged at 12,000 rpm for 20 min, and the supernatant was collected for experiments.

For western blotting, 10 mg of Lysate was separated by 10% sodium dodecyl sulfate polyacrylamide gel electrophoresis and transferred onto a nitrocellulose membrane (Invitrogen, Carlsbad, CA, USA). The membranes were blocked for 1 hour in TBST with 5% BSA at room temperature, and then incubated overnight at 4°C in TBST containing 5% BSA and following antibodies: c-Met antibody (Cat. Ab10728, abcam, China); β-actin antibody (Cat.ab3280, abcam, China). Membranes were washed 3 times in TBST, and incubated with a secondary antibody conjugated with horseradish peroxidase in TBST with 0.5% BSA for 2 hour at room temperature. After washed 3 times with TBST, bands were detected by chemiluminescence using Pierce ECL Western Blotting Substrate (cat. 32109, Pierce, USA). Intensity of the bands was detected and analyzed using Quantity One analyzing system (Bio-Rad, USA).

### Luciferase reporter assay

For validation of c-Met as a target genes of miR-34a in osteosarcoma cells, luciferase assay was performed as described previously [26].

### Target prediction

The prediction of miR-34a putative target genes was performed using bioinformatics methods based on sequence similarity between miRNAs and mRNAs. We queried TargetScan (http://www.targetscan.org/) [67], [68] and PicTar (http://pictar.mdc-berlin.de/) [69], [70]. Prediction algorithms change over time, and the analysis included here is from May 2011.

### Statistical Analysis

Each experiment was performed at least three times, and all values in the paper are reported as means±SD. Comparisons between groups were made with student's t-test. While statistical significances of mean difference among multiple groups were performed with analysis of variance (ANOVA) followed by post hoc Dunettes tests. And a P-value of less than 0.05 was accepted as statistically significant.

## Supporting Information

Figure S1 **DNA sequencing of plasmid pcDNA-miR34a.** The restriction enzyme cutting sites of BamH I and Hind III were underlined; the pri-miR-34a sequences were highlighted.
(PDF)

Figure S2 **Proliferation, migration and invasion assay of osteosacoma SOSP-9607 cells with transient transfection.** SOSP-9607 cells were transiently transfected with 50 nM of miR-34a mimics, mimics NC, miR-34a inhibitor and inhibitor NC (Genepharma, China), respectively. 48 h later, the proliferation (**A, B**), migration (**C, D**) and invasion (**E, F**) assay were performed. The results were presented as means±SD. *P<0.05, **P<0.01 (n = 3) were accepted as statistically significant.
(TIF)
