# Supplementary figures and images for: MicroRNA-34a Inhibits the Proliferation and Metastasis of Osteosarcoma Cells Both In Vitro and In Vivo
Source: PLoS One. 2012 Mar 21;7(3):e33778. doi: 10.1371/journal.pone.0033778 (PMC3310405; doi:10.1371/journal.pone.0033778)

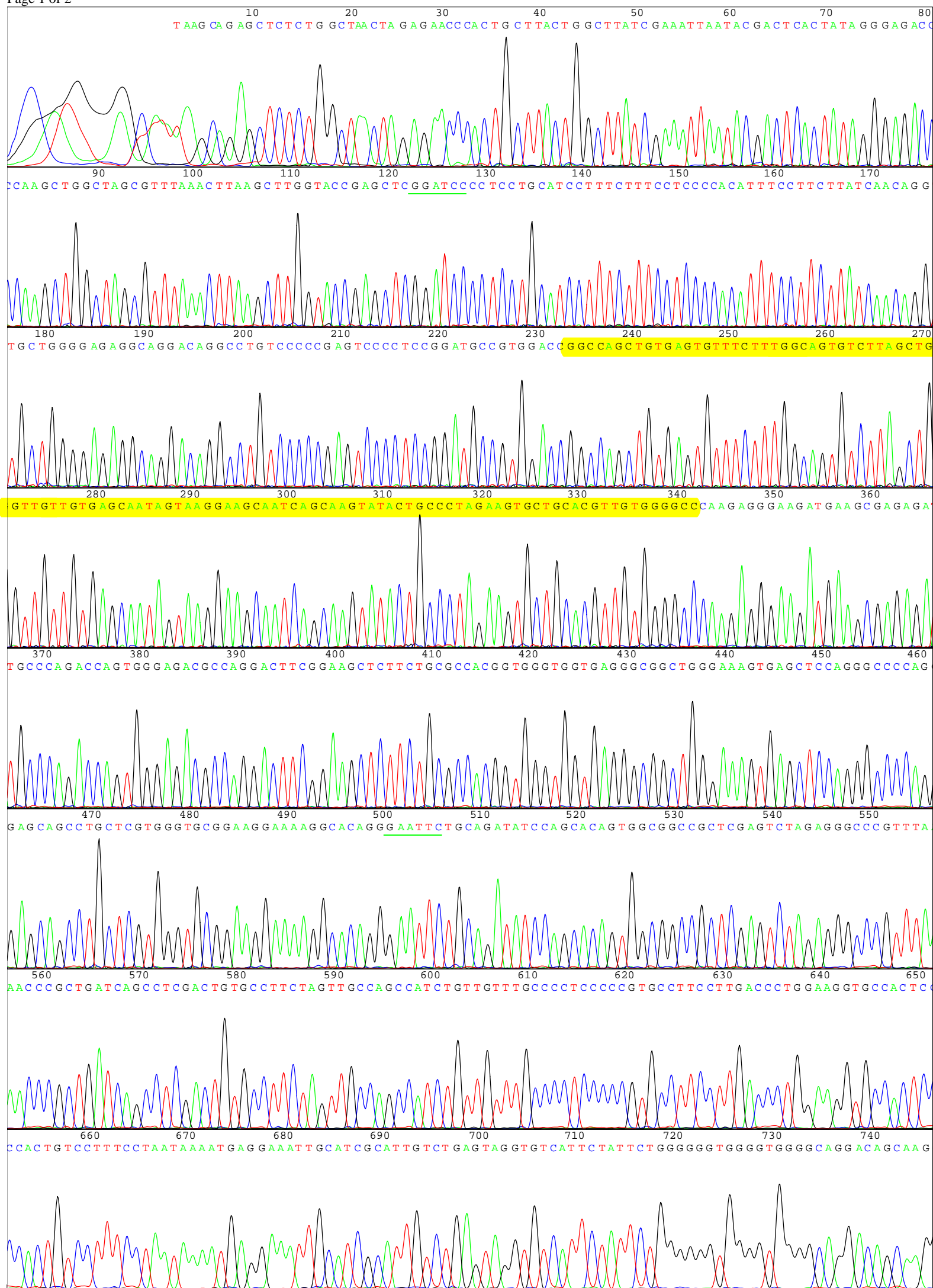

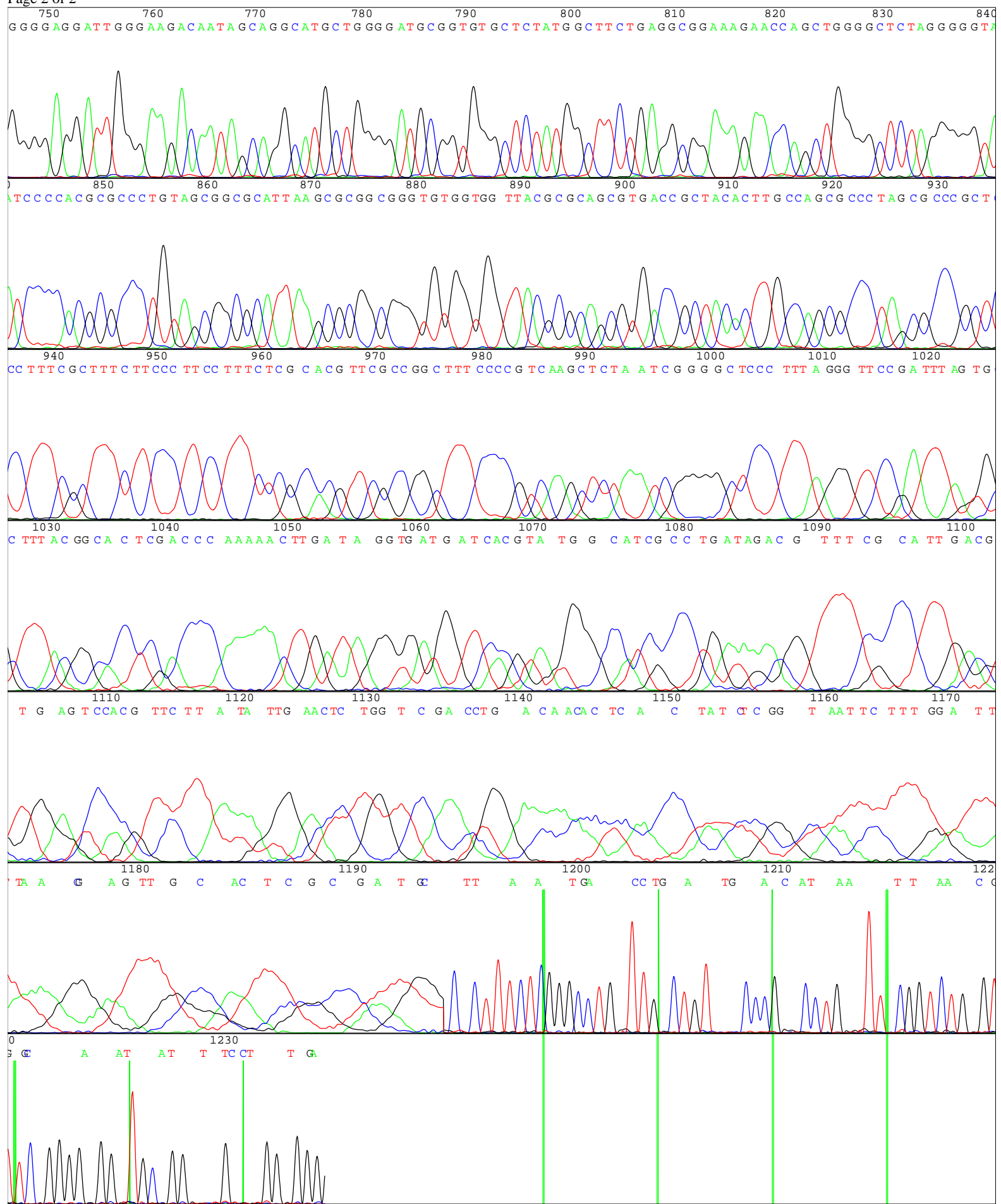

Supplement: Figure S1 — DNA sequencing of plasmid pcDNA-miR34a. The restriction enzyme cutting sites of BamH I and Hind III were underlined; the pri-miR-34a sequences were highlighted. (PDF) [file pone.0033778.s001.pdf]

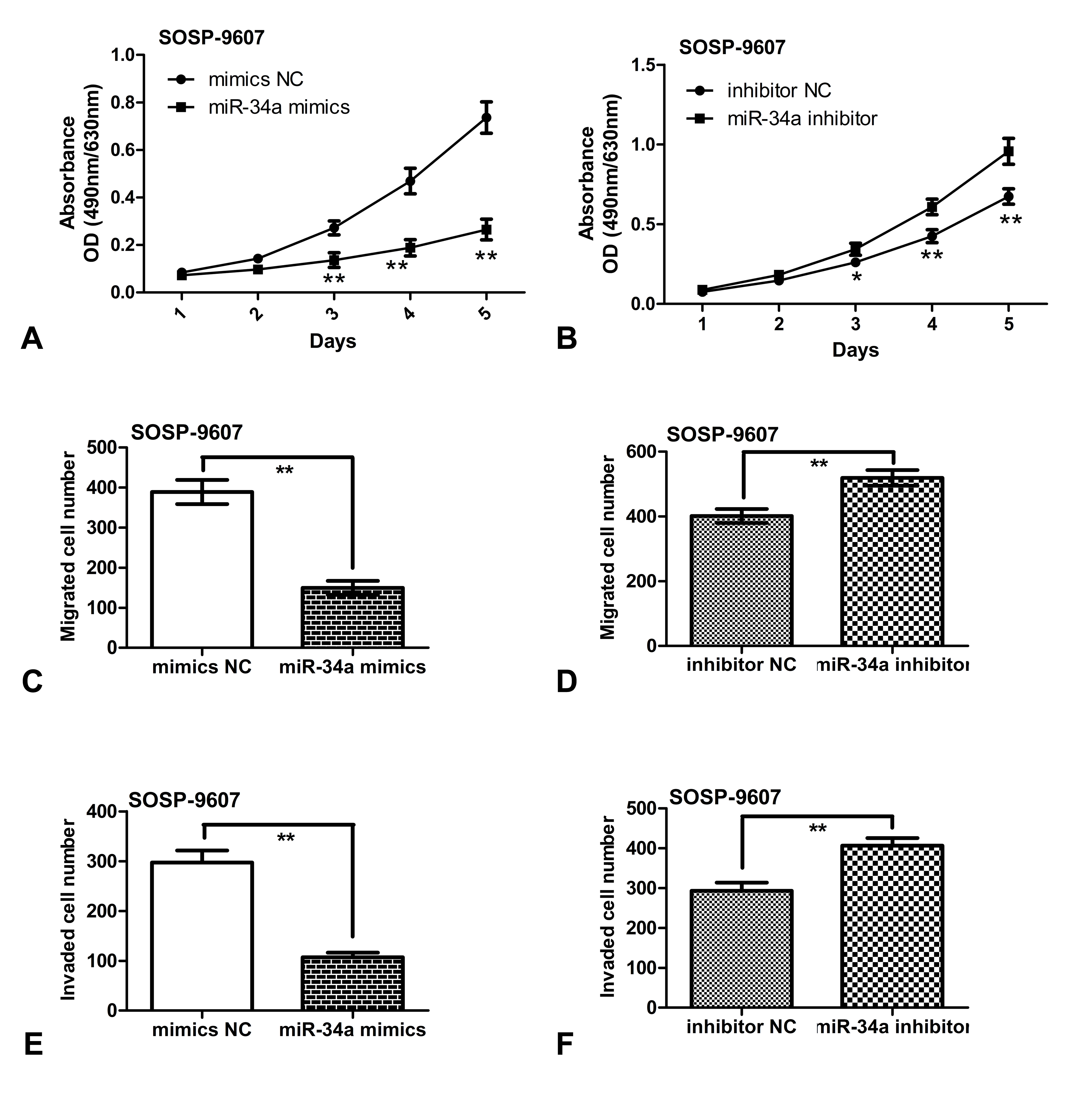

Supplement: Figure S2 — Proliferation, migration and invasion assay of osteosacoma SOSP-9607 cells with transient transfection. SOSP-9607 cells were transiently transfected with 50 nM of miR-34a mimics, mimics NC, miR-34a inhibitor and inhibitor NC (Genepharma, China), respectively. 48 h later, the proliferation (A, B), migration (C, D) and invasion (E, F) assay were performed. The results were presented as means±SD. *P<0.05, **P<0.01 (n = 3) were accepted as statistically significant. (TIF) [file pone.0033778.s002.tif]
